# Giant symptomatic serous cystadenoma mimicking carcinoma: A case report and literature review

**DOI:** 10.1016/j.ijscr.2019.05.042

**Published:** 2019-05-28

**Authors:** Lauren Pointer, Luke D. Rothermel, Carolina Strosberg, Daniel Anaya, Pamela Hodul

**Affiliations:** a University of South Florida, Gastroenterology, 12901 Bruce B. Downs Blvd., MDC 82, Tampa, FL, 33612, United States; b Moffitt Cancer Center, Department of Gastrointestinal Oncology, 12902 Magnolia Drive, Tampa, FL, 33612, United States; c Moffitt Cancer Center, Department of Anatomic Pathology, 12902 Magnolia Drive, Tampa, FL, 33612, United States

**Keywords:** Serous cystadenoma, Serous cystadenocarcinoma, Pancreatic cyst

## Abstract

**Introduction:**

Advanced imaging has led to an increase in the incidental diagnosis of pancreatic cysts. Serous cystadenomas (SCAs) account for nearly 30% of cases. These are typically considered benign lesions but up to 16% of cases are resected for aggressive behavior and symptoms.

**Presentation of case:**

A 64 year old female presented with a large incidental cyst in the pancreatic body. Pre-operative imaging conferred a diagnosis of SCA. After 2 years the cyst grew resulting in abdominal pain. Enlargement was associated with splenic vein occlusion and varices of the short gastric vessels. This change in behavior was concerning for malignant transformation. The cyst was resected by distal pancreatectomy and splenectomy. The patient recovered with minimal morbidity. Final pathology revealed a 15.5 × 10.3 × 8.5 cm SCA with negative margins.

**Discussion:**

In this case a patient presents with aggressive radiographic features and new symptoms suggesting malignant transformation of a previously diagnosed SCA on imaging. Although malignant variants can be diagnosed by findings of metastatic deposits at the time of surgery or as recurrence years later, histologic findings cannot discriminate benign from malignant potential. Preoperative imaging is challenging with erroneous characterization in nearly 60% of cases.

**Conclusion:**

Based on the low risk of malignancy, selective surgical resection for SCA appears warranted. Accepted indications for surgery include development of symptoms or concern for correct diagnosis in a medically fit individual. Routine resection for cysts >4 cm has been suggested however, prospective studies are needed to determine benefit over risk rationale.

## Highlights

• The diagnosis of serous cystadenoma is challenging.
• Surgery may be indicated for select serous cystadenoma.
• Serous cystadenocarcinoma is a rare entity and histologically indistinguishable from its benign counterpart.

## 1 Introduction

The detection of incidentally found pancreatic cysts has notably increased with the emergence and availability of advanced imaging. An extensive classification of cystic neoplasms of the pancreas and lesions that resemble them exists [1]. Many of these can present as large round masses including: mucinous cystic neoplasm (MCN), serous cystadenoma (SCA), solid pseudopapillary tumors, cystic pancreatic endocrine neoplasm, acinar cell carcinoma, and duplication cysts. Diagnosis is challenging. Imaging may not fit typical radiographic features and attempted aspirations of giant cysts are frequently acellular. Serous cystadenoma of the pancreas are considered benign lesions but up to 16% are resected for aggressive behavior or concern for malignancy [2]. Currently, no consensus exists regarding recommendations or indications for surveillance of SCA. Here we report a case of an enlarging suspected serous cystadenocarcinoma of the pancreas based on aggressive presentation. This work has been reported in accordance with the SCARE guidelines [3].

## 2 Presentation of case

**Fig. 1:**
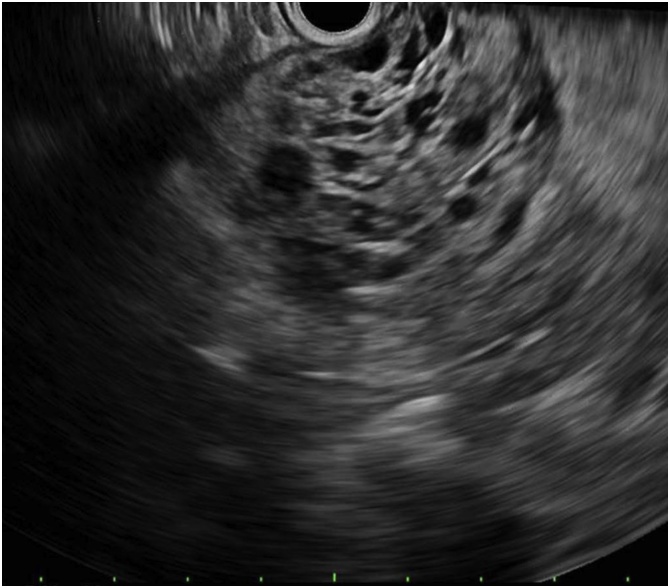
Endoscopic ultrasound. An anechoic, multicystic, septated and shadowing lesion suggestive of a cyst was identified in the pancreatic body and in the pancreatic tail. No communication with the pancreatic duct. Many thinly septated compartments, with no associated mass or internal debris within the fluid-filled cavity.

**Fig. 2:**
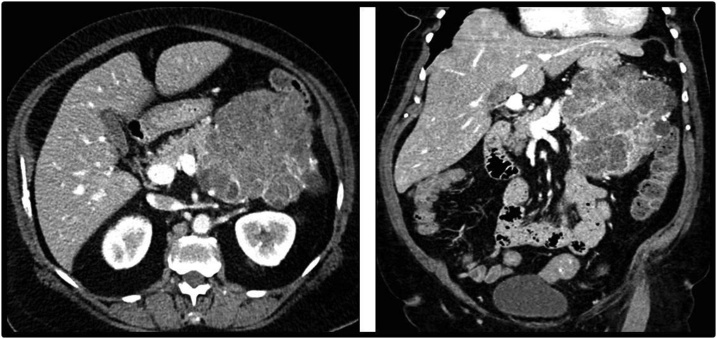
Computed tomography (CT) Imaging of Giant Serous Cystadenoma. Axial image, venous phase. Complex polycystic mass involving the entirety of the pancreatic body and tail measuring 12.4 x 11.0 cm in axial plane. Close proximity to surrounding visceral and vascular structures with concern for locally aggressive infiltration of this tumor. Coronal image, venous phase. Measuring 13.0 cm in craniocaudal dimension.

A 64 year old female was found to have an incidental pancreatic body cyst measuring 10 × 8 × 9 cm on computed tomography (CT) during work-up for pneumonia. An endoscopic ultrasound (EUS) described a honeycombed appearance with multiple small cysts suggestive of a SCA (Fig. 1). No aspiration was performed. Surveillance every 6 months was planned given the large size of the lesion. The cyst had been relatively stable until two years after diagnosis, when she presented to clinic with new onset diabetes mellitus and abdominal pain. CT at that time reported an increase in the size of the lesion to 12.4 × 11 x 13 cm involving the entire pancreatic body and tail with narrowing of the splenic vein and varices of the short gastric vessels (Fig. 2).

The patient’s case was presented at the multidisciplinary tumor board. Based on increased growth, aggressive radiographic findings, and new symptoms, surgical resection was recommended. The patient was taken to the operating room by a surgical oncologist who specializes in pancreatic resections. Upon midline laparotomy, the gastrocolic ligament was divided and the lesser sac was entered to expose the left upper quadrant mass. The mass extended from the diaphragm superiorly to the inferior mesenteric vein at the ligament of treitz inferiorly. The mass abutted the portal vein- splenic vein confluence and displaced the splenic flexure of the colon caudad. An antegrade pancreaticosplenectomy was performed. Retroperitoneal dissection revealed significant varices as well as infiltration of the colonic mesentery requiring resection. No metastatic disease was noted. The patient’s post-operative course was complicated by a grade A pancreatic leak which resolved within seven days post discharge. At nine months, the patient has no evidence for recurrent disease and her symptoms have completely resolved.

**Fig. 3:**
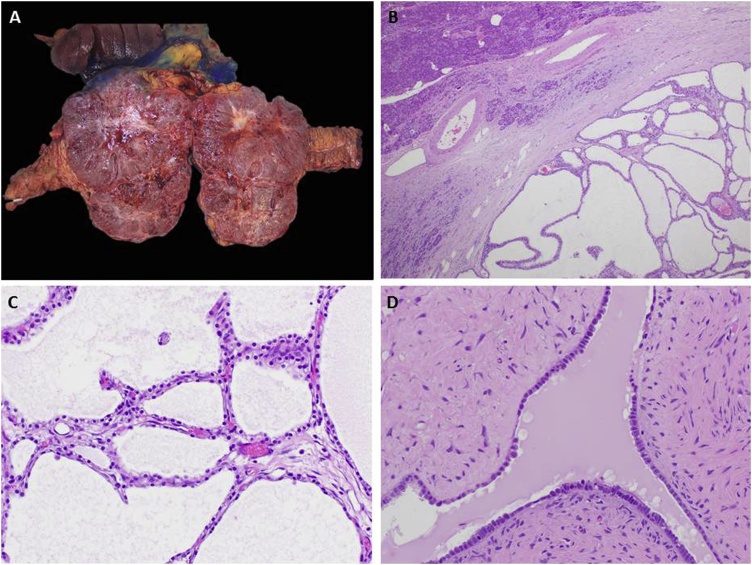
Serous cystadenoma. (**A**) Well-defined, polycystic mass involving the head of the pancreas. This gross appearance is seen as sponge-like or “honeycomb” on imaging. Note the central stellate scar and the delicate septa which can also be identified on imaging. (**B**) Low magnification shows a well circumscribed mass, adjacent to uninvolved pancreas (top left), with multiple, back to back, small thin-walled cysts filled with clear serous fluid. Interestingly, the cysts do not communicate with the pancreatic duct as seen in mucinous cystic neoplasms. (**C**) Rich capillary network contributes to the enhancement on CT imaging. (**D**) The epithelium lining the cystic spaces is composed of uniform columnar cells with pale cytoplasm rich in glycogen, without atypia or mitotic activity. This bland cytomorphology of a benign neoplasm can be deceiving in cases of serous cystadenocarcinoma, an extremely rare but morphologically indistinguishable tumor from serous cystadenoma.

Grossly the tumor measured 15.5 × 10.3 x 8.5 cm. It was encapsulated with fluid filled cysts ranging from 0.1 to 1.3 cm in dimension. Histologic findings confirmed a microcystic SCA (Fig. 3). Targeted next generation gene sequencing diagnosed a missense mutation in the von Hippel-Lindau (VHL) gene, p. H115y, a known mutation associated with SCAs. Margins were negative.

## 3 Discussion

SCA constitute up to 30% of pancreatic cystic neoplasms [2]. These lesions are predominantly encountered in middle- aged and older women, and are often discovered incidentally. Although predominantly considered benign, up to 30 cases of cystadenocarcinoma have been reported in the literature [4]. In addition, lesions in the head of the pancreas and those with large size may have a predilection for more aggressive behavior with adjacent organ or vascular involvement.^5^

Histologically, microcystic SCA are round, well circumscribed lesions most often identified in the tail of the pancreas. They contain multiple, small, thin-walled cysts filled with clear serous fluid which do not communicate with the pancreatic duct. The epithelium lining the cystic spaces is composed of uniform cells with pale cytoplasm rich in glycogen, without atypia or mitotic activity. Macrocystic and solid variants also exist. Histologically benign tumors are indistinguishable from malignant ones. The majority of cystadenocarcinoma are defined by findings of perineural or vascular invasion, or metastatic disease to the lymph nodes or liver at the time of resection, or as recurrence several years after the initial diagnosis [[4], [5], [6], [7]]. The metastatic deposits however, exhibit the same benign pathologic appearance as the original lesion [8].

Non-operative management with or without serial imaging is dependent on having a secure diagnosis. The radiographic diagnosis of SCA can prove challenging. This can lead to erroneous characterization and needless surgery in upwards of 60% of cases [9,10]. SCA are classified as (1) microcystic (2) oligocystic (3) solid-appearing or (4) mixed type. The microcystic type demonstrates a cluster of microcysts, the so called “honeycomb pattern” and is associated with a central scar in about 30% of cases [2]. Diagnosis can be difficult as the fibrosis within this subtype may mimic a mural nodule within an IPMN [11]. The oligocystic variant shares features with mucinous cystic neoplasms and branch duct IPMN. It is defined by a macrocystic component with lack of honeycomb pattern and ductal communication, and is frequently located in the head of the pancreas. The solid variant SCA is extremely rare and radiographically difficult to distinguish from the pancreatic endocrine neoplasm [11]. CT, magnetic resonance imaging (MRI), and EUS are the three most commonly used imaging techniques for revealing SCAs. CT alone is approximately 23% accurate at diagnosing SCA [5]. In contrast, diffusion weighted MRI demonstrates a 100% sensitivity and 97% specificity for differentiating SCAs from mucinous cysts [2]. EUS can provide information on cyst to duct communication and detailed description of the cystic components. However, EUS guided aspirates fail to provide a high level of diagnostic accuracy. In one study, serous epithelial cells were identified in < 20% of cases and gastrointestinal contaminating epithelium further contributed to difficulties in interpretation in over half of the cases [12].

As SCAs are generally believed to be a benign entity, surgery has been historically reserved for symptomatic patients, and management has been largely observation. Symptoms associated with SCAs are mostly related to mass effect or to infiltration of adjacent structures with abdominal pain and presence of a palpable mass most commonly described [13]. More contemporary reports argue for early surgical resection even in the absence of symptoms. Tseng et al recommend resection for SCA ≥ 4 cm, even in asymptomatic patients, based on their findings that larger tumors grow at faster rates (almost 2 cm/yr) and are more likely to cause symptoms [14]. Hwang suggests 5 cm as an indication for resection with consideration of minimally invasive surgical techniques [15]. Malleo et al report the fastest growth 7–10 years after diagnosis [16]. A more recent multicenter study failed to confirm these results. A growth rate of 6.2% per year or doubling time of 12 years was calculated for the non-resected SCA, while resected SCA grew faster (17% per year with doubling time of 4.5 years). These findings were independent of tumor size [17]. Similarly, in 2622 patients, the average growth rate was only 4 mm/year with stable size in 63% of patients [9]. Symptoms are more common when rapidly growing SCAs are left un-resected.

The prevalence of serous cystadenocarcinoma reported since 1989 is 3% [18]. Malignancy appeared in older patients with symptomatic lesions. The relationship between growth rate, size, and malignant potential is unknown [4]. In 25% the diagnosis of cancer was established only after growth of metachronous metastasis [4]. Furthermore, there are no deaths that are directly attributable to dissemination or malignant behavior of SCAs [19].

Greater use of radiography and advances in techniques has led to an increase in the incidental diagnosis of pancreatic cysts [20]. The diagnosis of SCA can be challenging and often leads to heightened anxiety for both the patient as well as the treating physician. While indications for surgery have been suggested, the literature is highly biased by reports of symptomatic patients with SCA who underwent resection. Universally acceptable guidelines for surgery include: patients with symptomatic SCA or indeterminate cysts in medically fit candidates. Despite recommendations for resection of smaller asymptomatic SCA, pancreatic resection at high volume institutions is notable for at least a 15% major and 30% minor risk of complications [8,14]. At least one comprehensive surveillance schema has been suggested [8]. In our case, the patient developed aggressive radiographic features including increase in growth and development of splenic vein compression with varices of the short gastric vessels over 12 months. In addition, she experienced new onset diabetes mellitus and increasing abdominal discomfort. These features were initially concerning for transformation of the serous cystadenoma to carcinoma leading to recommendations for surgical resection.

## 4 Conclusion

In conclusion, based on the low risk of malignancy, selective surgical resection for SCA appears warranted. Clear and universally accepted indications for surgery include associated symptoms, and/or a concern for the correct diagnosis in a medically fit individual [13]. Moderate indications based on the review of the literature may include an abrupt increase in growth rate or the exhibition of locally aggressive behavior. Prospective studies however, are further needed to determine whether routine resection of medium sized, asymptomatic SCA is beneficial over observation, assuming the morbidity associated with pancreatic surgery.

This research did not receive any specific grant from funding agencies in the public, commercial, or not-for-profit sectors.

## Conflicts of interest

The authors do not have any conflicts of interest to disclose related to this work.

## Funding

This research did not receive any specific grant from funding agencies in the public, commercial, or not-for-profit sectors.

## Ethical approval

According to the University of South Florida’s Institutional Review Board Policy and Procedure Manual “The USF IRB regards case reports or a limited case series as an educational activity, and therefore it is permissible under the Health Insurance Portability and Accountability Act (HIPAA) as a part of health care operations (45 CFR 164.501) when reviewing medical records. However, from both the Common Rule and the Privacy Rule perspective, a case series involving more than three (3) cases *does* meet the definition of research, and such research requires IRB approval.” Given this is a case report (that does not report on more than 3 patient cases), IRB approval was not needed.

## Consent

Written informed consent from the patient has been obtained.

## Author contribution

Pamela Hodul MD FACS: Conceptualization, review & editing, supervision.

Lauren Pointer MD: Data curation, writing.

Luke Rothermiel MD: Data curation, review & editing.

Carolina Strosberg MD: Data curation, writing.

Daniel Anaya MD FACS: Review & editing.

## Registration of research studies

N/A- this study did not require submission for approval from our local Institutional Review Board at the University of South Florida. Since, this met the definition of a case report as outlined in USF IRB’s policy and procedure manual as referenced above.

## Guarantor

Pamela J. Hodul MD, FACS.

## Provenance and peer review

Not commissioned, externally peer-reviewed.
